# Quality of life and general health of infertile women

**DOI:** 10.1186/s12955-017-0712-y

**Published:** 2017-07-12

**Authors:** Azam Namdar, Mohammad Mehdi Naghizadeh, Marziyeh Zamani, Farideh Yaghmaei, Mohammad Hadi Sameni

**Affiliations:** 1 Department of Health, School of Medicine, Jahrom University of Medical Sciences, Jahrom, Iran; 2 Noncommunicable Diseases Research Center, Fasa University of Medical Sciences, Fasa, Iran; 3 Department of Nutrition, School of Medicine, Jahrom University of Medical Sciences, Jahrom, Iran; 4 Department of Nursing, School of Nursing and Midwifery, Zanjan Branch, Islamic Azad University, Zanjan, Iran; 5 Department of English, School of Medicine, Jahrom University of Medical Sciences, Jahrom, Iran

**Keywords:** Infertility, Quality of life, General health, Rural population, Anxiety

## Abstract

**Background:**

Measuring the quality of life (QOL) is a benchmark in today’s world of medicine. The aim of the present study was to determine the general health and QOL of infertile women and certain affecting conditions.

**Methods:**

In a cross-sectional study, 161 infertile women referring to Dr. Rostami’s Infertility Center of Shiraz, Southern Iran, in 2013 were enrolled by the convenience sampling method. Data were collected via a socio-demographic, general health (GHQ28), and the QOL Questionnaire of Infertile Couples and analyzed using descriptive and analytical statistics.

**Results:**

According to 146 completely filled-out questionnaires, the mean age of the participants and their spouses were 29.4 ± 5.4 and 33.8 ± 5.8 years, respectively. Moreover, the general health of 57 (39%) patients was normal and that of 89 (61%) patients showed a degree of impairment. The scores for depression and physical symptoms were the highest and lowest, respectively. In addition, quite positive, positive, neutral, and negative specific QOL of infertile women were detected in 4 (2.8%), 72 (49.3%), 70 (47.9%), and 0 (0%) individuals, respectively. The total QOL scores had maximum correlation with GHQ anxiety (*r* = −0.596, *P* < 0.001) and general health scores had the highest correlation with physical QOL (*r* = −0.637, *P* < 0.001). The QOL was economically (*P* = 0.027), emotionally (*P* = 0.004), sexually (*P* = 0.017), physically (*P* = 0.037), and psychologically (*P* = 0.001) less for the women living in rural areas than other infertile women. However, university education (*P* = 0.015) and higher income per month (*P* = 0.008) had positive associations with QOL.

**Conclusion:**

General health of more than half of the infertile women indicated a degree of disorder. These women face the risk of anxiety, social dysfunction, and depression. Educational status, monthly income, and rural/urban residency are the major factors influencing the QOL.

## Background

Pregnancy and childbirth are valued roles for women in many developed and developing countries [1]. Infertility is defined as the failure to become pregnant despite regular sexual intercourse for one year [2]. It can cause considerable social distress and is accompanied by numerous psychological and social problems such as depression, anxiety, social isolation, and sexual dysfunction [3]. Infertile couples might experience psychological distress and suffer from an impaired health-related quality of life (QOL) [4]. It has been reported that infertility affects 10–15% of couples in industrialized countries in the age range of 18–45 years, many of whom are under excessive stress [5, 6]. There is a new definition in the literature for the fertility quality of life (FertiQOL), specifically evaluating the impact of fertility problems on various life dimensions [7]. Infertile women report poorer marital adjustment and QOL than the controls [8]. Moreover, men may experience less intercourse satisfaction, perhaps because of the psychological pressure of trying to conceive or the forced timing of intercourse around the woman’s ovulatory cycle [8]. However, it is still unclear whether this elevated level of distress occurs in all infertile couples, or whether certain sub-groups may have more problems. For example, the level of stress and changes in QOL may be related to socioeconomic status and other non-medical conditions. In addition, factors predicting QOL may vary in different infertile populations, genders, and ethnic backgrounds. Thus, the identification of factors associated with better or worse health-related QOL is vital for proposing and testing scientific interventions for infertile populations [9]. Nevertheless, no relevant data are available on such effects [10]. Infertility and mental health problems are related, and infertility is a different experience for women and men [10]. Furthermore, it has been reported that social factors influence attitudes about infertility and the lived experience of infertile individuals [2]. Therefore, the aim of the present study was to examine health-related QOL in infertile women referring to infertility clinics in Shiraz, Iran.

## Methods

This cross-sectional study was approved by the Ethics Committee of Jahrom University of Medical Sciences (Code No. IR.JUMS.REC.2012.009). The population consisted of all infertile women referring to Dr. Rostami’s Infertility Treatment Clinic in Shiraz in 2013. Out of 218 women who were registered at study time in the center, 161 were selected through convenience sampling, taking into consideration the inclusion criteria (women with primary infertility diagnosis who were willing to participate in the study and fill in the questionnaires). The data collection tool was a three-part questionnaire. The first part of the questionnaire dealt with demographic information (age, spouse’s age, sex, duration of marriage, level of education, occupation, income, place of residence, and history of pregnancy).

The second part was the Quality of Life Questionnaire for Infertile Couples, designed by Yaghmai and his colleagues [11]. The validity and reliability of this questionnaire were confirmed, with the Cronbach’s alpha of 0.81 and a test-retest reliability coefficient of 0.89 for the whole questionnaire [11]. This tool included 72 questions on seven divisions: physical, psychological, spiritual and religious beliefs, economic, sexual, emotional, and social. Each question had five choices: completely agree, agree, no idea, disagree, and completely disagree. Some questions dealt with positive and some others considered negative features in the study participants. The questions were scored as follows: 0 to 4 points were awarded for answers to questions dealing with positive features, from ‘completely agree’ to ‘completely disagree’, respectively. Similarly, 0 to 4 points were awarded for answers to questions dealing with negative features, from ‘completely agree’ to ‘completely disagree’, respectively. Then, the summed scores were converted to a percentage of the total score and interpreted in the following manner: ‘very negative’ QOL received less than 20% of the total score; ‘negative’ QOL was ≥20% but <40% of the total score; ‘neutral’ QOL was ≥40% but <60% of the total score; ‘positive’ QOL was ≥ 60% but <80% the total score; and ‘very positive’ QOL was ≥80% of the total score. In other words, the scores of QOL questionnaire in each area were between 0 and 100, and a higher score indicated a better QOL in that certain area. The third section contained the General Health Questionnaire (GHQ). The instrument utilized in this study was the 28-item General Health Questionnaire (GHQ-28) [12]. The GHQ-28 has four sub-scales, each consisting of 7 items. These scales which form the foundation of the GHQ include: (A) physical symptoms (1–7), (B) symptoms of anxiety (8–14), (C) social function (15–21), and (D) symptoms of depression (22–28). Each question was scored on a Likert scale (0–3). The lowest and the highest total scores were respectively 0 and 84, with lower scores signifying a more favorable public health [13]. The 28-question form had the advantage of being designed for all the members of the society [14]. Psychometric evaluation of GHQ-28 confirmed the reliability and validity of this questionnaire. Williams et al. used this tool for the meta-analysis of 43 studies and found a sensitivity of 84% and an average specificity of 84% [15]. This questionnaire was employed to assess the general health of participants. The number ‘22’ was considered as the best cut-off point in the 0-to-3 scoring method in the whole questionnaire [16]. This means that individuals who received a score lower than 22 were considered normal, and those with a score higher than 22 were considered abnormal. The interviewers were given necessary training on how to communicate with the participants and record the results in the questionnaire. Next, the researchers referred to the Infertility Clinic and after providing necessary information for the participants and obtaining written consent from them, completed the questionnaire by interviewing them. In case the participants preferred a self-report questionnaire, the duty was assigned to them.

After the completion of the questionnaires, analysis was done via IBM SPSS 18 (SPSS Inc., Chicago, Ill). Data were presented as mean, standard deviation, minimum, and maximum. QOL domain between the study groups was compared using independent t-test and ANOVA. Pearson’s correlation coefficient was used to compute the relationship between QOL domains. The *p*-value of less than 0.05 was considered statistically significant.

### Findings

#### Population characteristics

In this study, 161 married women referring to Dr. Rostami’s Infertility Clinic were interviewed. Only 146 responses to the questions were completely filled out and found acceptable for statistical and analytical interpretation.

The mean age of the patients was 29.4 ± 5.2 years, and the mean age of their spouses was 33.8 ± 5.8 years. The couples were married for an average of 6.6 ± 0.5 years. Of all these women, 101 (69.1%) were homemakers and 57 (39.0%) were employed. Fifty-eight infertile women (39.7%) and the same number of spouses had academic education. While 56 patients (38.3%) were from Shiraz, 67 (45.9%) were living in neighboring towns, and 23 (15.8%) came from rural areas.

#### General health (GHQ-28)

**Fig. 1:**
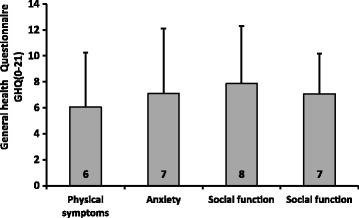
Sub- scales of GHQ (The health means more than it was then)

An evaluation of the general health status of infertile women indicated that the mean of the total score of GHQ-28 was 28.6 ± 13.0, with minimum and maximum values of 5 and 65, respectively. This revealed that the general health of 57 (39.0%) women was normal (0 to 22), and that of 89 women (61.0%) indicated a degree of disorder (23 or higher). The highest and lowest scores respectively belonged to the sub-scales of depression (the most disorders) and physical symptoms (the highest rate of health) (Fig. 1).

#### Quality of life (QOL)

**Fig. 2:**
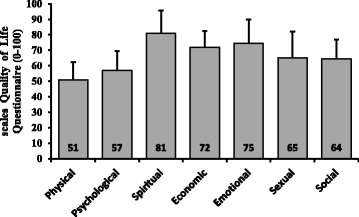
Sub-scales of the Quality of Life of Infertile Couples Questionnaire (score means better quality of life than it is later)

The evaluation of the quality of specific life of infertile women showed that the mean total score of the QOL questionnaire was 61.8 ± 2.9, with a minimum of 40.9 and a maximum of 88.5. Thus, QOL was quite positive in 4 individuals (2.8%), positive in 72 (49.3%), and neutral in 70 (47.9%). None of the patients had a negative QOL. The spiritual dimension showed the highest and the physical dimension revealed the lowest QOL scores (Fig. 2).

#### Correlation between quality of life and that of general health

Table 1 presents the correlation between dimensions of Specific Quality of Life Questionnaire and GHQ-28. Since the higher scores depict higher QOL and lower scores in the GHQ-28 point to greater health, most correlation coefficients in this table have negative values. The highest correlation is exhibited between the physical quality and physical symptoms of general health (*r* = −0.637, *p* < 0.001). Also, the psychological dimension of QOL demonstrated the highest correlation with the scores of general health anxiety (*r* = −0.538, *p* < 0.001). Nevertheless, the economic and emotional aspects of QOL, except for anxiety, did not show a meaningful correlation with other dimensions of general health. In addition, the social dimension of QOL had the highest correlation with the scores of social function of general health (*r* = −0.299, *p* < 0.001). The total score of QOL had the highest correlation with anxiety (*r* = −0.596, *P* < 0.001), and the total score of general health had the highest correlation with physical quality of life (*r* = −0.576, *P* < 0.001).

**Table 1:** Correlation between quality of life specialist and General health (GHQ-28) in infertile women

Quality of life	General health				
	Physical symptoms	Anxiety	Depression	Social function	Total score of the public health
Physical	−0.637^b^	−0.519^b^	−0.298^b^	−0.346^b^	−0.567^b^
Psychological	−0.337^b^	−0.538^b^	−0.236^b^	−0.307^b^	−0.453^b^
Spiritual	-0.169^a^	-0293^b^	−0.187^a^	−0.301^b^	−0.338^b^
Economic	−0.140	−0.178^a^	0.025	−0.013	−0.122
Emotional	−0.139	−0.237^b^	0.089	−0.123	−0.104
Sexual	−0.266^b^	−0.373^b^	−0.063	−0.164^a^	−0.280^b^
Social	−0.230^b^	−0.340^b^	−0.032	−0.299^b^	−0.286^b^
Total score of the Quality of Life	−0.479^b^	−0.596^b^	−0.222^a^	−0.394^b^	−0.543^b^

^a^Significantly between (0.05 to 0.01)

^b^Significantly lower than 0.01

#### Relationship between QOL and patient characteristics

Assessment of the relationships between different dimensions of QOL and demographic specifications of the population revealed that the variables of wife’s age, husband’s age, age difference between spouses, and duration of marriage had no correlation with any aspects of QOL and general health (data not shown). However, the sub-scale of physical QOL was the highest in women with a university education (*P* = 0.022). This physical quality was the lowest for women who had a monthly income of less than IRR 2,000,000 (*P* = 0.034) and those living in rural areas (*P* = 0.037). The psychological and mental QOL was the highest in women with an academic education (*P* = 0.004) and the employed ones (*P* = 0.026). This value was the lowest in women with a monthly income of less than IRR 2,000,000 (*P* = 0.008) and those living in rural areas (*P* = 0.001) (Table 2). The QOL was economically (*P* = 0.027), emotionally (*P* = 0.004), and sexually (*P* = 0.017) lower in women living in rural areas compared to other infertile women. Furthermore, the social sub-scale of QOL was the highest in women with a university education (*P* = 0.015). This dimension was the lowest in women with a monthly income of less than IRR 2,000,000 (*P* = 0.008) and those living in rural areas (*P* < 0.001). The spiritual dimension of QOL was not associated with any of the above variables (*P* > 0.05) (Table 2).

**Table 2:** Relationship between sub-scales quality of life and some of the variables in infertile women

Variable		Quality of life							
		Number	Physical	Psychological	Spiritual	Economic	Emotional	Sexual	Social
Female education	> diploma	88	49.3 (11.2)	54.4 (11.2)	81.0 (12.9)	72.1 (10.7)	72.8 (15.9)	65.2 (16.6)	62.1 (12.1)
	college	58	53.6 (10.6)	60.5 (13.6)	80.9 (16.9)	72.2 (10.2)	77.0 (14.0)	64.8 (17.6)	67.5 (12.1)
	*P* value		0.022	0.004	0.938	0.983	0.104	0.888	0.010
Spouse education	< diploma	88	50.6 (10.9)	56.4 (12.2)	80.8 (14.8)	72.8 (10.6)	72.7 (16.3)	66.8 (17.0)	63.7 (13.0)
	College	58	51.7 (11.5)	57.4 (12.9)	81.3 (14.4)	71.1 (10.3)	77.4 (13.2)	62.5 (16.7)	65.3 (11.4)
	*P* value		0.561	0.653	0.832	0.359	0.072	0.133	0.465
Wife’s occupation	House wife	101	50.4 (10.2)	55.2 (11.2)	82.0 (13.1)	72.5 (10.3)	74.5 (15.4)	65.0 (16.3)	63.0 (12.0)
	Employed	45	52.5 (13.0)	60.2 (14.4)	78.9 (17.5)	71.3 (11.0)	74.3 (15.0)	65.2 (18.5)	67.3 (12.7)
	*P* value		0.308	0.026	0.237	0.539	0.898	0.942	0.053
Spouse occupation	Stable income	59	52.2 (10.8)	57.6 (12.8)	81.9 (15.0)	72.9 (10.6)	77.5 (15.9)	64.5 (17.6)	66.4 (12.6)
	Seasonal income	87	50.3 (11.3)	56.3 (12.3)	80.4 (14.4)	71.6 (10.4)	72.5 (14.5)	65.5 (16.5)	62.9 (12.1)
	*P* value		0.298	0.544	0.551	0.454	0.056	0.733	0.095
Monthly income (Rls)	<2,000,000	17	45.4 (12.6)	49.9 (11.7)	76.3 (18.5)	71.8 (7.7)	71.2 (17.5)	67.1 (16.0)	603 (14.0)
	200–8,000,000	104	51.2 (10.5)	56.6 (12.1)	82.8 (12.6)	71.1 (10.2)	74.9 (15.0)	64.2 (16.9)	64.6 (11.8)
	>8,000,000	25	54.4 (11.4)	62.2()12.6	76.6 (18.2)	76.6 (12.3)	75.6 (15.0)	62.7 (18.0)	66.3 (13.6)
	*P* value		0.034	0.008	0.161	0.061	0.607	0.643	0.301
Place of residence	Shiraz city	56	52.6 (11.9)	56.6 (11.0)	78.3 (14.6)	69.8 (11.6)	72.3 (15.3)	60.7 (17.0)	62.5 (11.5)
	Neighboring towns	67	51.7 (10.6)	59.7 (13.0)	83.9 (14.2)	74.7 (9.3)	78.8 (15.0)	69.4 (16.5)	66.9 (13.9)
	Rural	25	45.7 (9.4)	48.9 (11.5)	79.0 (14.9)	70.4 (9.5)	67.8 (12.3)	63.0 (15.9)	61.4 (8.2)
	*P* value		0.037	0.001	0.086	0.027	0.004	0.017	0.073

Assessment of the total score of QOL showed that educated women (*P* = 0.015) and those with higher incomes (*P* = 0.008) had better QOL, and those living in rural areas had the lowest QOL (*P* < 0.001). General health was not associated with any of the above variables (*P* > 0.05) (Table 3).

**Table 3:** Comparison of quality of life and General health with some of the variables in infertile women

Variable		Number	Quality of life	General health
Female education	<diploma	88	60.3 (8.0)	29.6 (14.0)
	college education	58	63.9 (9.5)	26.4 (10.5)
	*P* value		0.015	0.180
Spouse education	<diploma	88	61.5 (9.1)	29.6 (13.6)
	college education	58	62.2 (8.3)	26.4 (11.4)
	*P* value		0.623	0.180
Wife’s occupation	House wife	101	61.1 (7.8)	28.2 (13.5)
	Employed	45	63.3 (10.6)	28.9 (11.5)
	*P* value		0.157	0.804
Spouse occupation	Stable income	59	62.7 (8.8)	26.5 (10.7)
	Seasonal income	87	61.1 (8.8)	29.5 (13.9)
	*P* value		0.292	0.199
Monthly income (Rls)	<2,000,000	17	56.1 (8.6)	31.9 (15.0)
	200–8,000,000	104	62.0 (8.2)	28.4 (12.4)
	>8,000,000	25	64.9 (9.7)	25.7 (13.3)
	*P* value		0.008	0.351
Place of residence	Shiraz city	56	61.0 (8.0)	28.0 (12.6)
	Neighboring towns	67	64.4 (9.0)	28.0 (12.9)
	Rural	25	56.4 (7.2)	30.3 (14.0)
	*P* value		<0.001	0.763

Results as mean (standard deviation) is shown. Significantly from the comparison between the two groups (education, employment, insurance and pregnancy), and compared between groups using *t*-test (income and place of residence) is using analysis of variance. Where significant changes have shown notable

## Discussion

In the present study, for the first time, the QOL of the infertile women who attended an infertility clinic in Shiraz was evaluated. None of the patients had a negative QOL. The spiritual dimension showed the highest and the physical dimension revealed the lowest QOL scores. The total QOL score had the highest correlation with anxiety, and the total score of general health had the highest correlation with physical QOL. The QOL for women living in rural areas was economically, emotionally, sexually, physically, and psychologically lower compared to other infertile women. Also, university education and higher monthly income had positive associations with QOL. The roles of education, income, and urban residency found in this study as the major factors affecting the QOL of infertile women can be explained using their compensatory roles in both financial and emotional aspects. Indeed, high levels of education, and especially university education, residency in cities, and higher monthly income may fill the place of a child to a certain extent. This can prevent the decrease of QOL in such infertile women in comparison to those with lower incomes and education and those living in rural areas. Social factors can influence infertility, and it is reasonable to expect that the prevalence of mental disorders in infertile individuals should vary cross-culturally [2]. It has been reported that infertile women differ from the fertile ones in terms of some psychological properties such as narcissism, dimensions of attachment style, and uses of defense mechanism [17]. There is a two-way relationship that infertile and depressed women are less likely to initiate fertility treatments [18], and infertile patients who receive infertility treatments may have negatively-affected QOL [19]. Moreover, it is confirmed that infertility is associated with decreased scores of QOL domains, mostly affecting mental health, vitality, and emotional behavior, as well as psychological, environmental, physical, and social functioning [20]. Indeed, infertility as a public health problem reduces some special aspects of QOL through negative psychosocial and cultural consequences, and induces depression, anxiety, social isolation and deprivation, marital instability, loss of self-esteem, loss of gender identity, loss of control, and feeling of self-blame and guilt [21–24]. It has been reported that socioeconomic status, mental health, religiosity, physical health, and future imagining are important dimensions of QOL among postmenopausal infertile Iranian women [1]. A study conducted on 112 women treated for infertility in Taiwan found anxiety (23%), major depression (17%), and dysthymic disorder (10%) [25]. Furthermore, in another study on 141 infertile and 65 fertile Korean women, infertile women had higher scores of depression, anxiety, and stress [26]. Dural et al. also reported that infertile patients with a high QOL had lower degrees of depression and anxiety and vice versa [7]. Findings similar to our result were observed in a recent study conducted in China by Xiaoli et al. Based on their results, infertile women had lower QOL scores in spirituality, religion, personal beliefs, self-esteem, and financial resources [27]. Finally, according to a meta-analysis evaluating 14 related studies published between January 1980 and July 2009, infertile women had significantly lower QOL scores on mental health, social functioning, and emotional behavior, compared with fertile controls [19]. We found that the psychological dimension of QOL had a greater correlation with scores of general health anxiety. It has been mentioned that infertility is associated with high rates of anxiety symptoms in Finland and USA [10, 28]. As seen, infertility especially in women is accompanied by several general health problems, which decreases QOL. One of these problems is anxiety disorder, which is composed of a group of mental disorders characterized by the feelings of anxiety and fear.

There are several reports worldwide on infertility and anxiety. For instance, 52–83.8% of infertile women in China [29], 33% in Hong-Kong [30], 86.6% in Iran [31], 67% in Spain [32], and 24.9% in the Netherlands, Belgium, and France [33] showed anxiety symptoms. A case-control study confirmed that primary infertile women aided by reproductive assistance technology display lower scores on mental and physical dimensions, vitality, social functioning, emotional functioning, and mental health than fertile female controls [34]. It is clear that the treatment of infertility can affect QOL. However, this must be performed scientifically, as presented in infertility clinics. Porat-Katz et al. reported that users of complementary medicine reported increased relational and lower social QOL, increased use of psychosocial support, and favorable healthy lifestyle habits [35].

## Conclusion

Our findings showed that general health of more than half of the infertile women indicated a degree of disorder who face the risks of anxiety, social dysfunction, and depression. Educational status, monthly income, and rural/urban residency are the major factors affecting QOL. To better understand such effects, performing case-control studies with larger sample sizes in different regions is highly recommended. In addition, the psychological distress and QOL of the infertile Iranian women, as detected in this study, seemed to need psychological interventions.

## References

[1] Direkvand-Moghadam A, Delpisheh A, Montazeri A, Sayehmiri K (2016). Quality of life among Iranian infertile women in postmenopausal period: a cross-sectional study. J Menopausal Med.

[2] Sezgin H, Hocaoglu C, Guvendag-Guven ES (2016). Disability, psychiatric symptoms, and quality of life in infertile women: a cross-sectional study in Turkey. Shanghai arc psychiatry.

[3] Baghiani Moghadam MH, Aminian AH, Abdoli AM, Seighal N, Falahzadeh H, Ghasemi N (2011). Evaluation of the general health of the infertile couples. Iran J Reprod Med.

[4] Rashidi B, Montazeri A, Ramezanzadeh F, Shariat M, Abedinia N, Ashrafi M (2008). Health-related quality of life in infertile couples receiving IVF or ICSI treatment. BMC Health Serv Res.

[5] Lo SS, Kok WM (2016). Sexual functioning and quality of life of Hong Kong Chinese women with infertility problem. Hum Fertil (Camb).

[6] Mosher WD, Pratt WF (1991). Fecundity and infertility in the United States: incidence and trends. Fertil Steril.

[7] Dural O, Yasa C, Keyif B, Celiksoy H, Demiral I, Yuksel Ozgor B, Gungor Ugurlucan F, Bastu E (2016). Effect of infertility on quality of life of women: a validation study of the Turkish FertiQoL. Hum Fertil.

[8] Monga M, Alexandrescu B, Katz SE, Stein M, Ganiats T (2004). Impact of infertility on quality of life, marital adjustment, and sexual function. Urology.

[9] Kedem P, Mikulincer M, Nathanson YE, Bartoov B (1990). Psychological aspects of male infertility. Br J Med Psychol.

[10] Klemetti R, Raitanen J, Sihvo S, Saarni S, Koponen P (2010). Infertility, mental disorders and well-being--a nationwide survey. Acta Obstet Gynecol Scand.

[11] Yaghmaei F, Mohammadi S, Alavimajd H. Developing “Quality of Life in Infertile Couples Questionnaire” and Measuring its Psychometric Properties. J Reprod Infertil. 2009;10(2):137–43. [Persian]

[12] Goldberg DP, Hillier VF (1979). A scaled version of the general health questionnaire. Psychol Med.

[13] Noorbala AA, Mohammad K. The validation of general health questionnaire- 28 as a psychiatric screening tool. Hakim Health Sys Res. 2009;11(4):47–53. [Persian]

[14] Shakeri J, Hossieni M, Golshani S, Sadeghi K, Fizollahy V (2006). Assessment of general health, stress coping and marital satisfaction in infertile women undergoing IVF treatment. J Reprod Infertile.

[15] Goldberg D (1989). Screening psychiatric disorders. In: Williams p, Wilkinson G, editors. The Scape of epidemiological psychiatry.

[16] Palahang H (1999). Prevalence of mental disorders in the internal ward of Kashani hospital, Shahrekord, 1999. J Shahrekord Univ Med Sci.

[17] Poddar S, Sanyal N, Mukherjee U (2014). Psychological profile of women with infertility: a comparative study. Ind Psychiatry J.

[18] Crawford NM, Hoff HS, Mersereau JE (2017). Infertile women who screen positive for depression are less likely to initiate fertility treatments. Hum Reprod.

[19] Chachamovich JR, Chachamovich E, Ezer H, Fleck MP, Knauth D, Passos EP (2010). Investigating quality of life and health-related quality of life in infertility: a systematic review. J Psychosom Obstet Gynaecol.

[20] Chachamovich J, Chachamovich E, Fleck MP, Cordova FP, Knauth D, Passos E (2009). Congruence of quality of life among infertile men and women: findings from a couple-based study. Hum Reprod.

[21] van Balen F, Bos HM (2009). The social and cultural consequences of being childless in poor-resource areas. Facts views vision ObGyn.

[22] Maroufizadeh S, Karimi E, Vesali S, Omani Samani R (2015). Anxiety and depression after failure of assisted reproductive treatment among patients experiencing infertility. Int J Gynaecol Obstet.

[23] Maroufizadeh S, Ghaheri A, Amini P, Samani RO (2017). Psychometric properties of the fertility quality of life instrument in infertile iranian women. Int J Fertil Steril.

[24] Cousineau TM, Domar AD (2007). Psychological impact of infertility. Best Pract Res Clin Obstet Gynaecol.

[25] Chen TH, Chang SP, Tsai CF, Juang KD (2004). Prevalence of depressive and anxiety disorders in an assisted reproductive technique clinic. Hum Reprod.

[26] Chi HJ, Park IH, Sun HG, Kim JW, Lee KH (2016). Psychological distress and fertility quality of life (FertiQoL) in infertile Korean women: the first validation study of Korean FertiQoL. Clin Exp Reprod Med.

[27] Xiaoli S, Mei L, Junjun B, Shu D, Zhaolian W, Jin W, Ju Q, Wanli S, Huali Z, Li J (2016). Assessing the quality of life of infertile Chinese women: a cross-sectional study. Taiwan J Obstet Gynecol.

[28] King RB (2003). Subfecundity and anxiety in a nationally representative sample. Soc Sci Med.

[29] Lok IH, Lee DTS, Cheung LP, Chung WS, Lo WK, Haines CJ (2002). Psychiatric morbidity amongst infertile Chinese women undergoing treatment with assisted reproductive technology and the impact of treatment failure. Gynecol Obstet Investig.

[30] Lu Y, Yang L (1995). Lu G: [mental status and personality of infertile women]. Zhonghua Fu Chan Ke Za Zhi.

[31] Ramezanzadeh F, Aghssa MM, Abedinia N, Zayeri F, Khanafshar N, Shariat M, Jafarabadi M (2004). A survey of relationship between anxiety, depression and duration of infertility. BMC Womens Health.

[32] Guerra D, Llobera A, Veiga A, Barri PN (1998). Psychiatric morbidity in couples attending a fertility service. Hum Reprod.

[33] Oddens BJ, den Tonkelaar I, Nieuwenhuyse H (1999). Psychosocial experiences in women facing fertility problems-a comparative survey. Hum Reprod.

[34] El Kissi Y, Amamou B, Hidar S, Ayoubi Idrissi K, Khairi H, Ali BBH (2016). Quality of life of infertile Tunisian couples and differences according to gender. Int J Gynecol Obstet.

[35] Porat-Katz A, Paltiel O, Kahane A, Eldar-Geva T (2016). The effect of using complementary medicine on the infertility-specific quality of life of women undergoing in vitro fertilization. Int J Gynaecol Obstet.

